# Supplementary material for: Nanopore-Sequencing Characterization of the Gut Microbiota of Melolontha melolontha Larvae: Contribution to Protection against Entomopathogenic Nematodes?
Source: Pathogens. 2021 Mar 25;10(4):396. doi: 10.3390/pathogens10040396 (PMC8067285; doi:10.3390/pathogens10040396)
Supplement: Supplementary file 1 [file pathogens-10-00396-s001.pdf]

Supplementary Material

Journal name: Pathogens

Special Issue: Issue "Harnessing Host-Pathogen-Microbiota Interactions for Sustainable Disease Management"

Title:

Nanopore-sequencing characterization of the gut microbiota of *Melolontha melolontha* larvae: contribution to protection against entomopathogenic nematodes?

Author names:

Ewa Sajnaga[1]*, Marcin Skowronek[1], Agnieszka Kalwasińska[2], Waldemar Kazimierczak[1], Karolina Ferenc[3], Magdalena Lis[1], Adrian Wiater[4]

Author affiliations:

[1] Laboratory of Biocontrol, Application and Production of EPN, Centre for Interdisciplinary John Paul II Catholic University of Lublin, Konstantynów 1J, 20-708 Lublin, Poland;

[2] Department of Environmental Microbiology and Biotechnology, Nicolaus Copernicus University in Torun, Lwowska 1, Torun, 87-100, Poland;

[3] Department of Large Animal Diseases with Clinic, Institute of Veterinary Medicine, Warsaw University of Life Sciences, Nowoursynowska 100, 02-797 Warsaw, Poland

[4] Department of Industrial and Environmental Microbiology, Institute of Biological Sciences, Maria Curie-Skłodowska University, ul. Akademicka 19, 20-033 Lublin, Poland.

***Corresponding authorat:** Centre for Interdisciplinary Research, John Paul II Catholic University of Lublin, ul. Konstantynów1J, 20-708 Lublin, Poland;

E-mail address: ewa.sajnaga@kul.pl (E. Sajnaga)

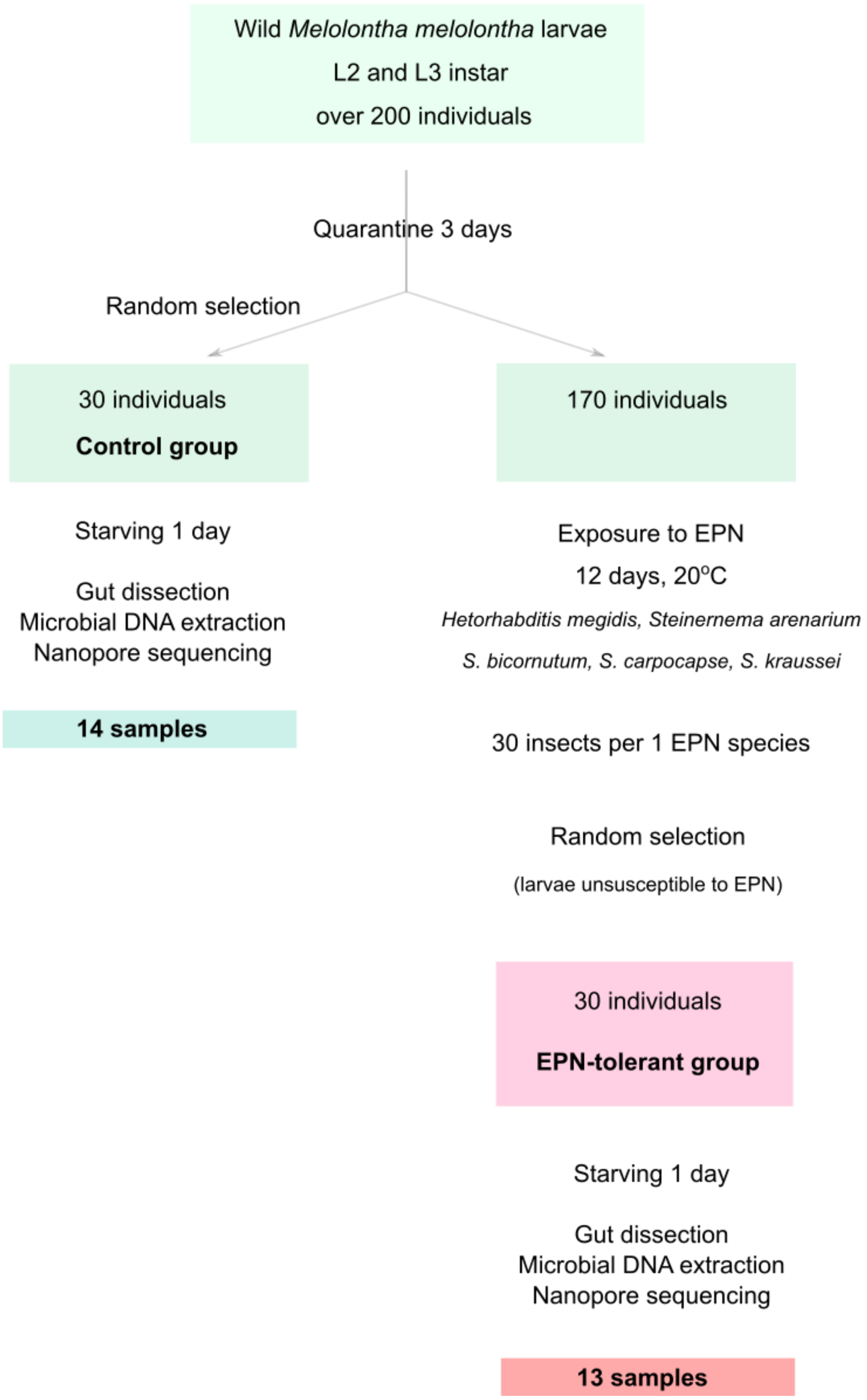

Figure S1. Schematic diagram of the course of the experiment

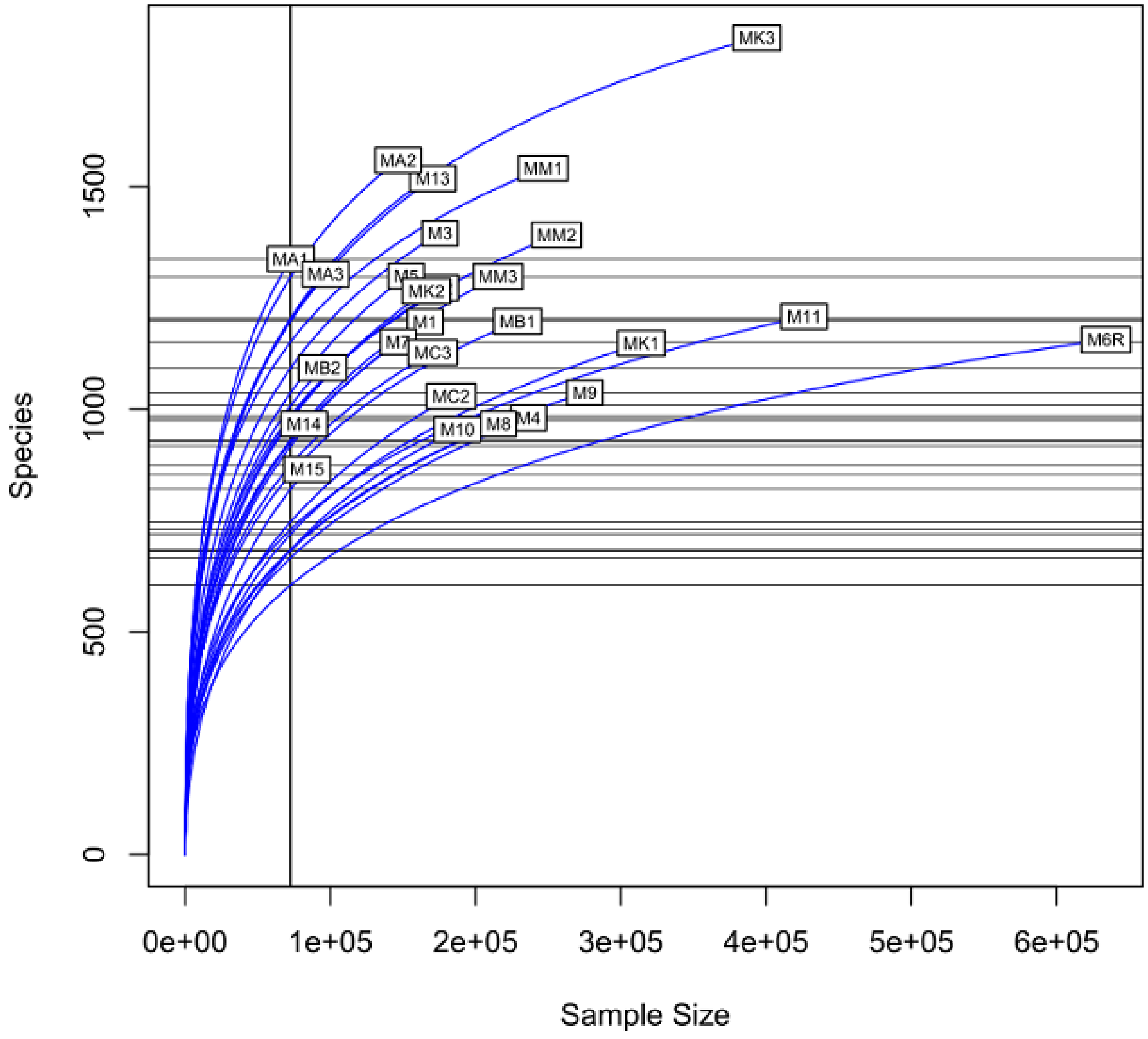

Figure S2. Rarefraction curves from nanopore sequencing of the 16S rRNA gene

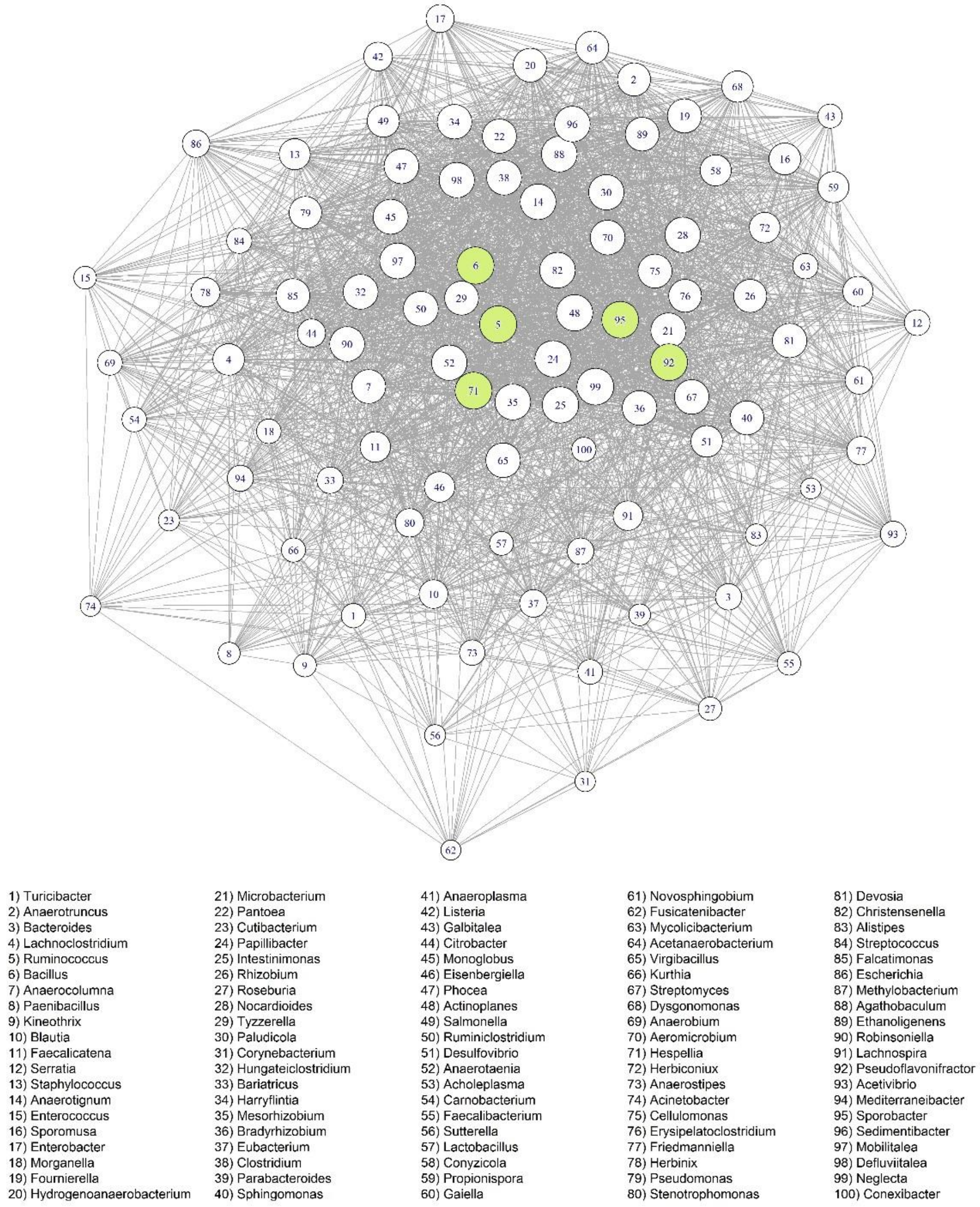

Figure S3. Mirobiome network of the midgut bacterial genera found in the control group of *M. melolontha* larvae

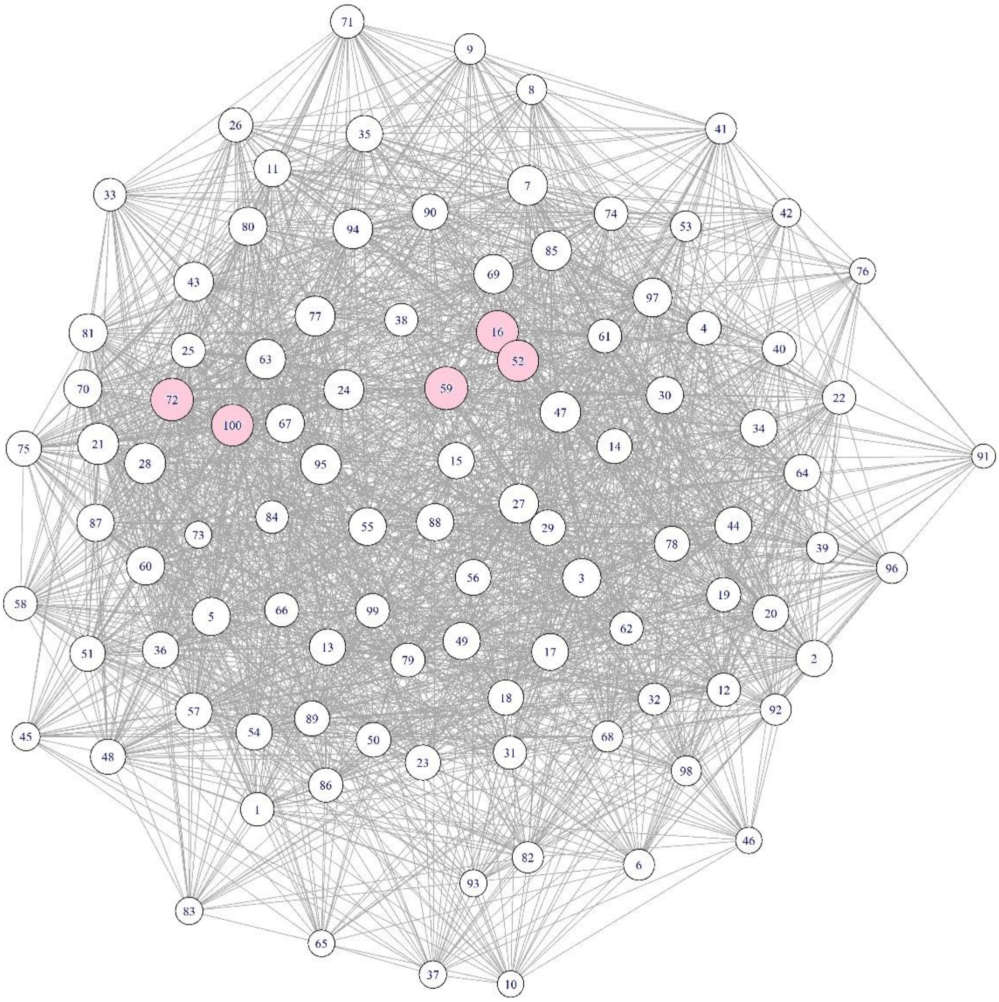

1) Turicibacter
2) Anaerotruncus
3) Bacteroides
4) Lachnoclostridium
5) Ruminococcus
6) Bacillus
7) Anaerocolumna
8) Paenibacillus
9) Kineothrix
10) Blautia
11) Faecalicatena
12) Serratia
13) Staphylococcus
14) Anaerotignum
15) Enterococcus
16) Sporomusa
17) Enterobacter
18) Morganella
19) Fournierella
20) Hydrogenoanaerobacterium
21) Microbacterium
22) Pantoea
23) Cutibacterium
24) Papillibacter
25) Intestinimonas
26) Rhizobium
27) Roseburia
28) Nocardioides
29) Tyzzerella
30) Paludicola
31) Corynebacterium
32) Hungateiclostridium
33) Bariatricus
34) Harryflintia
35) Mesorhizobium
36) Bradyrhizobium
37) Eubacterium
38) Clostridium
39) Parabacteroides
40) Sphingomonas
41) Anaeroplasma
42) Listeria
43) Galbitalea
44) Citrobacter
45) Monoglobus
46) Eisenbergiella
47) Phocea
48) Actinoplanes
49) Salmonella
50) Ruminiclostridium
51) Desulfovibrio
52) Anaerotaenia
53) Acholeplasma
54) Carnobacterium
55) Faecalibacterium
56) Sutterella
57) Lactobacillus
58) Conyzicola
59) Propionispora
60) Gaiella
61) Novosphingobium
62) Fusicatenibacter
63) Mycolicibacterium
64) Acetanaerobacterium
65) Virgibacillus
66) Kurthia
67) Streptomyces
68) Dysgonomonas
69) Anaerobium
70) Aeromicrobium
71) Hespellia
72) Herbiconiux
73) Anaerostipes
74) Acinetobacter
75) Cellulomonas
76) Erysipelatoclostridium
77) Friedmanniella
78) Herbinix
79) Pseudomonas
80) Stenotrophomonas
81) Devosia
82) Christensenella
83) Alistipes
84) Streptococcus
85) Falcatimonas
86) Escherichia
87) Methylobacterium
88) Agathobaculum
89) Ethanoligenens
90) Robinsoniella
91) Lachnospira
92) Pseudoflavonifractor
93) Acetivibrio
94) Mediterraneibacter
95) Sporobacter
96) Sedimentibacter
97) Mobilitalea
98) Defluviitalea
99) Neglecta
100) Conexibacter

Figure S4. Mirobiome network of the midgut bacterial genera in the EPN-resistant group of *M. melolontha* larvae

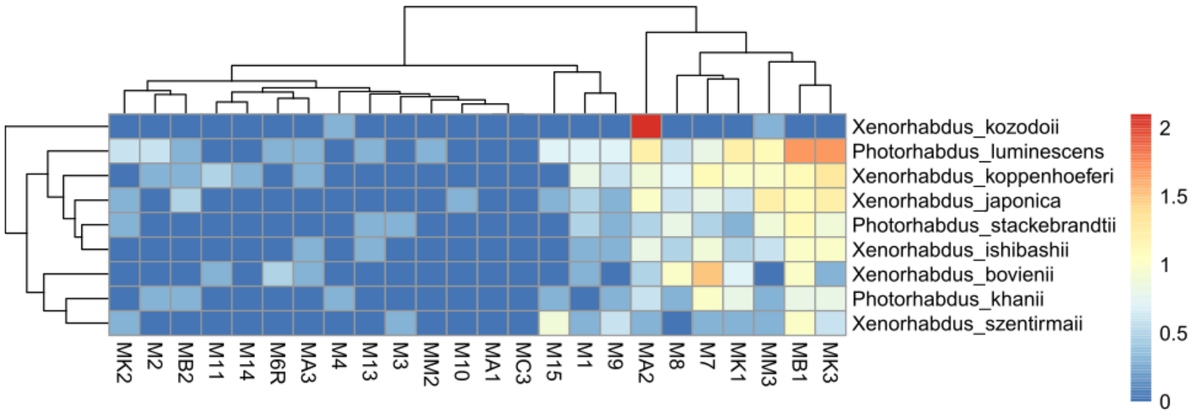

Figure S5. Heatmap showing the relative abundance of *Xenorhabdus* and *Photorhabdus* spp. detected in the midguts of all tested individuals

Table S1a. NGS sequencing statistics and bacterial diversity indicators for the midgut microbiota of the control group of *M. melolontha* larvae

Sample name	Nematode exposure	Sampling site	Developmental stage of larva	Total number of bacterial reads	Classification rate genus level (%)	Classification rate species level (%)	Sobs	Shannon	Simpson	Evenness
M1	No	ZF	L2	176,411	93.7	69.2	865	3.4	0.91	0.14
M2	No	ZF	L2	185,004	95.0	86.8	928	4.2	0.94	0.14
M3	No	PF	L2	182,284	96.3	86.9	1,045	3.8	0.86	0.12
M4	No	PF	L2	255,949	92.3	88.3	640	3.7	0.92	0.14
M5	No	PF	L2	160,319	95.0	86.6	934	4.4	0.97	0.14
M6R	No	KG	L2	675,822	93.8	90.8	595	3.7	0.93	0.15
M7	No	KG	L2	165,194	88.8	88.7	855	4.3	0.97	0.14
M8	No	KG	L3	227,905	94.7	88.7	643	3.6	0.92	0.14
M9	No	KG	L3	303,510	90.7	89.5	662	4.1	0.97	0.15
M10	No	KF	L3	191,361	97.9	93.5	635	3.9	0.91	0.14
M11	No	KF	L3	456,497	93.4	91.6	724	4.3	0.97	0.15
M13	No	KF	L3	181,730	93.9	84.7	1,113	4.5	0.97	0.14
M14	No	KF	L2	87,463	93.7	89.6	801	4.3	0.97	0.14
M15	No	KF	L2	86,641	96.9	89.8	720	3.6	0.90	0.14

Table S1b. NGS sequencing statistics and bacterial diversity indicators for the midgut microbiota of the EPN-resistant group of *M. melolontha* larvae

Sample name	Nematode exposure	Sampling site	Developmental stage of larva	Total number of bacterial reads	Classification rate genus level (%)	Classification rate species level (%)	Sobs	Shannon	Simpson	Evenness
MA1	Yes	KF	L2	77,759	93.5	86.2	1,165	4.9	0.98	0.14
MA2	Yes	KF	L2	158,972	92.3	82.7	1,217	4.8	0.97	0.14
MA3	Yes	KF	L2	104,015	93.0	84.5	1,083	5.1	0.99	0.14
MB1	Yes	KF	L2	249,252	91.8	86.8	813	3.9	0.94	0.14
MB2	Yes	KF	L2	99,846	94.8	87.7	896	4.4	0.96	0.14
MC2	Yes	KF	L2	190,503	96.1	94.1	701	2.4	0.65	0.10
MC3	Yes	KF	L3	177,462	96.1	92.7	829	4.7	0.98	0.15
MK1	Yes	KF	L2	326,700	96.1	88.9	691	3.6	0.93	0.14
MK2	Yes	KF	L2	173,240	95.9	89.6	924	4.1	0.96	0.14
MK3	Yes	KF	L3	422,249	93.2	87.1	1,201	4.7	0.98	0.14
MM1	Yes	KF	L2	259,941	94.9	80.1	1,112	4.8	0.98	0.14
MM2	Yes	KF	L2	275,099	93.0	86.6	965	4.2	0.96	0.14
MM3	Yes	KF	L3	222,727	96.7	91.9	874	3.9	0.91	0.13

Table S2. Proportions of the top twenty most abundant shared bacterial genera detected in the midgut of *M. melolontha* larvae

	M10	M11	M13	M14	M15	M1	M2	M3	M4	M5	M6R	M7	M8	M9	MA1	MA2	MA3	MB1	MB2	MC2	MC3	MK1	MK2	MK3	MM1	MM2	MM3
Turicibacter	76.14	9.73	10.88	0.33	0.46	0.06	0.01	0.17	0.06	2.26	0.27	0.23	0.06	3.40	0.08	0.50	0.58	1.85	0.02	5.14	47.18	11.63	1.00	2.73	0.50	0.28	38.88
Bacteroides	0.01	5.08	0.09	1.36	10.59	5.95	2.10	4.08	0.84	10.79	21.58	3.42	4.02	2.81	11.24	4.19	0.34	1.33	15.75	0.44	0.49	19.62	5.79	9.78	0.17	0.84	0.36
Anaerotruncus	0.16	8.06	6.37	8.29	2.36	0.11	0.82	2.64	5.25	2.62	7.14	5.80	20.73	1.78	0.05	0.61	2.06	0.88	0.86	55.27	3.59	0.07	0.32	3.65	6.57	4.19	0.13
Lachnoclostridium	0.02	2.17	1.83	11.36	0.73	0.52	22.34	3.29	23.08	1.89	3.83	8.80	1.01	1.57	0.55	0.32	1.24	6.14	1.26	0.96	1.59	0.61	0.14	0.70	2.05	1.41	0.06
Ruminococcus	0.02	3.47	1.27	2.57	0.58	0.44	3.11	0.86	4.51	1.61	2.07	4.18	2.55	5.53	0.51	0.32	0.96	2.28	1.78	5.24	1.06	7.05	5.82	4.13	0.64	3.91	0.23
Anaerocolumna	0.01	0.50	0.55	0.31	0.01	0.13	2.52	0.80	8.84	0.53	4.31	4.03	0.59	1.17	0.01	0.02	2.22	10.03	0.68	1.96	4.38	0.21	0.02	1.42	0.15	1.70	0.07
Bacillus	2.24	0.74	9.64	2.51	6.23	17.72	1.18	1.04	0.16	0.69	0.15	0.65	0.37	1.43	0.97	0.31	2.74	1.48	1.23	0.27	2.41	1.89	0.45	2.26	1.72	0.19	2.64
Kineothrix	0.01	0.17	0.64	2.99	0.59	0.96	3.88	0.27	3.71	0.69	0.15	1.27	0.23	6.38	0.48	0.09	0.90	5.81	2.29	0.14	0.69	1.46	0.17	0.49	0.54	10.93	0.22
Faecalicatena	0.00	0.31	0.13	0.53	0.09	0.18	3.78	0.15	2.57	0.26	0.48	2.11	0.17	7.65	0.03	0.05	0.47	3.00	2.46	0.25	1.09	1.76	0.24	0.33	0.05	12.25	0.24
Blautia	0.01	0.60	0.33	1.24	1.03	1.64	2.89	0.73	3.66	1.14	1.20	1.20	0.61	2.63	1.63	1.24	1.54	8.88	2.95	0.43	0.99	0.32	4.39	0.74	0.26	1.56	0.03
Paludicola	0.01	6.97	0.37	0.62	0.05	0.05	0.33	0.23	0.61	1.26	6.66	0.70	1.20	0.21	0.00	0.05	0.31	0.44	0.06	0.17	1.33	0.03	0.26	0.18	0.15	0.24	0.00
Paenibacillus	1.00	1.09	0.58	0.79	0.76	0.10	0.05	35.98	0.30	0.81	0.28	0.50	0.19	0.23	0.74	0.33	0.52	0.11	0.06	0.20	0.21	0.17	0.08	0.30	0.32	0.45	0.26
Serratia	0.01	0.06	0.01	0.01	0.05	0.05	0.03	0.10	0.00	0.93	0.32	9.06	8.23	0.02	0.36	0.19	2.06	14.48	0.21	0.00	0.00	0.16	0.02	0.20	0.03	0.00	1.81
Staphylococcus	0.51	0.18	0.03	0.11	0.37	14.86	1.77	0.04	0.01	1.57	0.01	0.06	0.13	1.26	0.19	0.02	0.46	1.53	1.75	0.12	0.32	4.32	2.60	2.26	0.06	0.00	0.10
Hydrogenoanaerobacterium	0.03	2.08	2.15	1.56	0.26	0.01	0.22	0.42	1.35	0.73	2.83	1.38	2.84	0.72	0.02	0.12	0.49	0.34	0.27	4.59	1.71	0.01	0.04	0.85	0.43	0.96	0.00
Enterobacter	0.01	0.00	0.02	0.00	0.05	0.35	0.68	0.03	0.00	0.02	0.01	0.05	0.02	0.95	0.09	2.95	0.00	0.31	0.03	0.00	0.06	1.22	2.59	2.74	0.00	0.01	17.03
Sporomusa	0.10	1.84	0.22	2.91	2.74	0.04	0.20	0.27	1.08	0.65	1.50	1.35	3.59	1.20	0.14	0.16	1.11	2.99	0.29	2.26	1.29	0.41	0.14	0.98	0.52	1.39	0.04
Fournierella	0.05	3.37	1.16	1.67	0.53	0.08	0.33	0.70	1.76	1.10	0.75	1.74	4.47	0.59	0.11	0.30	0.65	0.42	0.60	1.67	1.10	0.10	1.05	0.86	0.86	0.88	0.03
Papillibacter	0.01	1.53	0.11	1.87	0.50	0.08	0.17	0.55	1.54	0.50	0.91	3.04	1.44	7.19	0.01	0.03	1.25	0.26	0.17	0.14	0.24	2.04	0.13	0.86	0.42	0.39	0.02
Enterococcus	0.17	0.06	0.06	0.07	0.00	7.38	0.01	0.21	0.04	0.05	0.02	0.04	13.74	0.03	0.35	0.01	0.04	4.77	0.84	0.02	0.79	0.81	0.02	0.10	0.01	0.12	1.10

Table S3. Relative abundance of bacterial species exhibiting antagonistic activity against *Xenorhabdus* and *Photorhabdus* entomopathogens detected in the midgut of the EPN-resistant and control groups of insects

Species name	Abundance (mean in %) and 95% confidence interval	
	Control larvae	EPN-resistant larvae
Serratia liquefaciens	0.141 [0-0.325]	0.080 [0-0.179]
Acinetobacter calcoaceticus	0.046 [0-0.114]	0.073 [0.001-0.146]
Citrobacter murliniae	0.029 [0-0.061]	0.036 [0-0.077]
Pseudomonas chlororaphis	0.003 [0-0.01]	<0.001
Chryseobacterium lathyri	<0.001	<0.001
Antagonistic species in total	0.22 [0-0.47]*	0.19 [0.05-0.33]*

*no statistically significant differences between the groups

Table S4. Relative abundance of *Xenorhabdus* and *Photorhabdus* spp. detected in the midgut of the EPN-resistant and control groups of insects

Taxname	Abundance (mean in %) and 95% confidence interval		
	Control insects	EPN-resistant insects	All insects
Photorhabdus	0.003 [0.001-0.005]*	0.008 [0.002-0.014]*	0.005 [0.002-0.009]
P._luminescens	n.d.	n.d.	0.003 [0.001-0.004]
P._stackebrandtii	n.d.	n.d.	0.001 [0-0.001]
P._khanii	n.d.	n.d.	0.001 [0-0.001]
Xenorhabdus	0.008 [0-0.015]*	0.016 [0-0.034]*	0.012 [0.003-0.021]
X._kozodoii	n.d.	n.d.	0.003 [0-0.01]
X._koppenhoeferi	n.d.	n.d.	0.001 [0.001-0.002]
X._bovienii	n.d.	n.d.	0.001 [0-0.003]
X._japonica	n.d.	n.d.	0.001 [0-0.002]
X. ishibashii	n.d.	n.d.	0.001 [0-0.001
X._szentirmaii	n.d.	n.d.	0.001 [0-0.001]

Main abundant species (>0.001%) are indicated

*no statistically significant differences between the groups

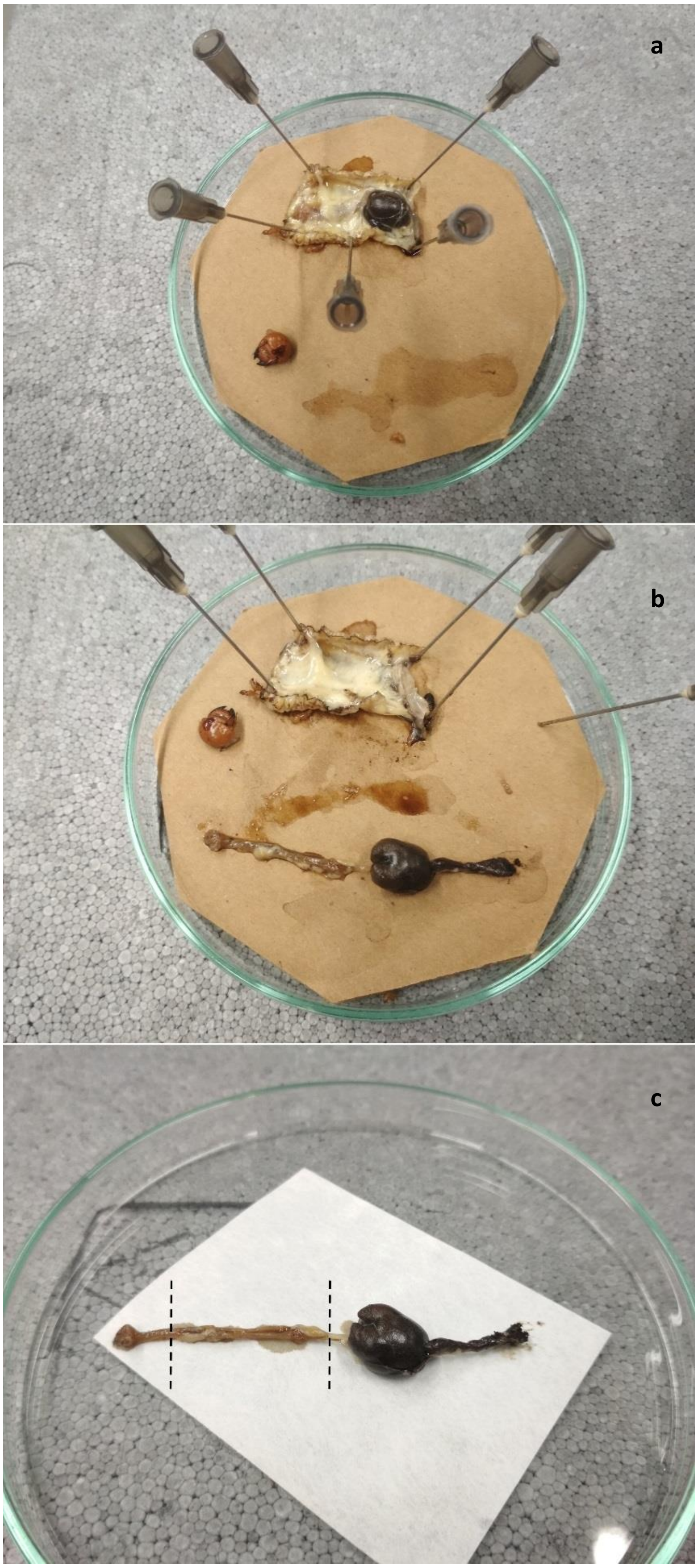

Phot. S1. Stages of *M. melolontha* larva section (a, b) and an overview of the whole larval gut (c). The midgut is visible between the dashed lines.
